# Predicting the Airborne Transmission of Measles: Impact of Indoor Carbon Dioxide (CO2) Levels and Mitigation Strategies

**DOI:** 10.7759/cureus.64882

**Published:** 2024-07-19

**Authors:** Narumichi Iwamura, Kanako Tsutsumi

**Affiliations:** 1 Department of Nephrology, Yamaguchi Red Cross Hospital, Yamaguchi, JPN

**Keywords:** immunocompromised hosts, covid-19, basic reproduction number, mask efficacy, infection probability, wells–riley model, indoor air quality, co2 monitoring, airborne transmission, measles virus

## Abstract

Background: Measles is a highly contagious cause of febrile illness typically seen in young children. It is transmitted primarily through respiratory droplets and small-particle aerosols and can remain viable in the air. Despite the availability of an effective vaccine, measles remains a major global issue, particularly in regions with low vaccination rates.

Aim: This study aimed to quantify the airborne transmission risk of the measles virus in various indoor environments.

Methods: Using indoor carbon dioxide (CO_2_) levels, we estimated the probability of airborne transmission and the basic reproduction number (Ro) in four hypothetical indoor scenarios, including restaurants, mass gathering events, homes, and business meetings, based on the modified Wells-Riley model.

Results: The relationship between airborne transmission rates and indoor CO_2 _concentrations was visualized, with and without mask usage. Without masks, at an indoor CO_2_ concentration of 1,000 ppm, the airborne transmission rates were high in homes (100.0%) and business meetings (100.0%) and moderate in restaurants (45.6%) and live events (30.6%). By contrast, the Ro was high in audience-participatory live events (60.9%) and restaurants (13.2%), indicating a higher risk of cluster infections.

Discussion and conclusion: In all indoor environmental scenarios, a positive linear relationship was found between the risk of airborne transmission and indoor CO_2_ levels. The risk of airborne transmission varied significantly across scenarios, which was influenced by various parameters, such as mask usage, quality of ventilation, conversation, and exposure duration. This model suggests that the risk of airborne transmission of measles can be easily predicted using a CO_2_ meter.

## Introduction

Measles, caused by the measles virus, is a highly communicable viral ailment that presents substantial public health challenges globally. Despite the availability of a potent vaccine, measles continues to be a predominant cause of morbidity and mortality among young children, particularly in regions with suboptimal vaccination coverage. Based on the World Health Organization (WHO), the incidence of measles in the Western Pacific Region increased by 255% from 2022 to 2023, with 1,080, 1422, and 5,044 cases in 2021, 2022, and 2023, respectively [1]. The vaccination coverage in several countries in the Western Pacific Region declined during the COVID-19 pandemic. Moreover, the ongoing measles outbreaks in the Philippines and persistent endemic measles transmission in Malaysia pose a threat of measles resurgence in the Western Pacific Region from 2024 to 2025. Based on the Ministry of Health, Labour, and Welfare, 744 cases were reported in Japan in 2019. The annual number of cases remained below 10 during the COVID-19 pandemic from 2020 to 2022, but it increased to 28 in 2023. As of March 22, 2024, more than 20 cases have been confirmed in at least eight prefectures [2].

A critical factor contributing to the high transmissibility of measles is its mode of transmission. Measles predominantly propagates through airborne transmission, transpiring when an infected individual coughs or sneezes, disseminating virus-laden droplets into the air. These droplets can remain suspended in the air for up to two hours, thereby posing an infection risk to individuals who inhale them [3]. The basic reproduction number (Ro) for measles is between 12 and 18, rendering it one of the most infectious diseases in humans [4]. Therefore, to prepare for the next potential measles pandemic, enhancing herd immunity through vaccination and recognizing scenarios with a high probability of airborne transmission is crucial. Emphasis should be placed on measures, such as wearing masks and maintaining good ventilation, to mitigate the risk of infection.

In our preceding research, we evaluated the risk of airborne transmission of the severe acute respiratory syndrome coronavirus 2 (SARS-CoV-2) Omicron variant within indoor environments, utilizing carbon dioxide (CO_2_) concentrations as a surrogate indicator [5]. This study introduced an enhanced version of the Wells-Riley equation, traditionally utilized for estimating the probability of airborne infection by integrating measurements of indoor CO_2_ levels. This study introduced an enhanced version of the Wells-Riley equation, traditionally utilized for estimating the probability of airborne infection, by integrating measurements of indoor CO_2_ levels. This study applied and adapted the modified Wells-Riley model to quantify the airborne transmission risk of SARS-CoV-2 based on the detected CO_2_ concentrations in indoor air. The efficacy of the model was validated by analyzing three case scenarios within a hospital, each depicting varying ventilation conditions. The results substantiated the model’s utility as a robust tool to assess infection probabilities in real-world scenarios, bearing significant implications for public health and safety protocols.

Concurrently, this study aimed to evaluate the airborne transmission rate of the measles virus across a range of nonhospital environments by utilizing the modified Wells-Riley model. This study had various scenarios, including restaurants, live events, home, and business meetings. We also aimed to provide graphical representations to facilitate the use of CO_2_ concentration meters for straightforward predictions of measles virus airborne transmission rates and to identify situations with heightened transmission risks.

## Materials and methods

Sample and data

We did not collect samples or perform statistical analysis.

Measures

We did not perform any actual measurements of data, including CO_2_ concentrations.

Models and data analysis

We utilized the modified Wells-Riley model, which used indoor CO_2_ concentrations for estimating airborne infection probability and the basic Ro across 10 distinct scenarios: restaurants, live events, buses, and business conferences. Table 1 shows the parameters for each scenario. 

**Table 1:** Key parameters for each scenario

Scenario	Exposure time (hours)	Number of individuals staying in the room	Conversation
Restaurants	0.5	30	Yes
Live events	2.0	200	Yes
Home	24	4	Yes
Business meetings	0.5	2	Yes

Furthermore, we derived a modified Wells-Riley model using indoor CO_2_ concentrations for estimating airborne infection probability, consistent with our previous research. Riley et al. originally developed the Wells-Riley equation to estimate airborne infection probability in indoor environments [6]:

$PI= \frac{C'}{S}= 1-e^{-\frac{Iqpt}{Q}}$　　　　　(1)

where P_I_ = infection probability (−), C’ = number of susceptible individuals that were infected (−), S = number of susceptible individuals (−), I = number of infectious individuals (−), q = generation rate of infectious quanta (/h), p = pulmonary ventilation rate of a person (m^3^/h), t = exposure time (h), and Q = rate of room ventilation with clean air (m^3^/h),　

The Wells-Riley model necessitates the assumption of well-mixed air within a room, ensuring a uniform distribution of aerosols, and focusing on airborne transmission instead of droplet or contact transmission. Additionally, the model does not consider the activation state of infectious particles. Due to the SARS-CoV-2 pandemic, the use of face masks became ubiquitous within the Japanese population. The presence or absence of a mask on both infectious and susceptible individuals, including the type of mask, such as surgical or N95, plays a critical role in determining the probability of infection. Thus, Dai and Zhao introduced a revised version of the Wells-Riley model that incorporates these significant variables [7].

$PI= \frac{C'}{S}= 1-e^{-\frac{Iqpt}{Q}\left ( 1-\eta I \right )\left ( 1-\eta S \right )}$　　　　　(2)

where η_I_ is the exhalation filtration efficacy (−), and η_S_ is the respiration filtration efficacy (−).

The Wells-Riley model often poses application challenges due to the requirement of data on room ventilation rate with clean air (Q), a value traditionally inferred from the performance metrics of ventilation fans and air conditioning systems. However, this ventilation rate is frequently subject to variations induced by factors such as the opening of doors and windows and the velocity and direction of external wind, thereby complicating the ability to obtain precise estimates and subsequently limiting the model’s applicability. Therefore, this study estimated room ventilation rates by considering indoor CO_2_ concentrations and emission rates. The Seidel formula, presented herein, elucidates the relationship between room ventilation rates and indoor CO_2_ concentrations:

$Co\cdot Q'\cdot dt+M\cdot dt-C\cdot Q'\cdot dt=V\cdot dC$

$C=Co+\left ( Cs-Co \right )e^{-\frac{Q}{V}t}+\frac{M}{Q}\left ( 1-e^{-\frac{Q}{V}t} \right )$　　　　　(3) 

In this context, C represents the indoor CO_2 _concentration (ppm), Co​ is the atmospheric CO_2_ concentration (ppm), Cs is the initial indoor CO_2_ concentration (ppm), Q′ denotes the rate of room ventilation with clean air per person (m^3^/h/person), V stands for the room volume (m^3^), T is the time (h), and M is the CO_2_ emission rate of an individual (m^3^/h/person).

When the CO_2_ emission rate of an individual (M) remains constant and sufficient time has passed, the indoor CO_2_ concentration (C) will reach equilibrium. As a result, the room ventilation rate with clean air per person (Q′) can be calculated using the following formula:

$C=Co+\frac{M}{Q'}$

$Q'=\frac{M}{C-Co}$　　　　　(4)

The clean air ventilation rate for the room (Q, m^3^/h) is defined as follows:

$Q=nQ'$　　　　　(5)

where n is the number of individuals staying in the room (person).

The value of Q can be determined by substituting Equation (5) into Equation (4) as shown below:

$Q=n\frac{M}{C-Co}$　　　　　(6)

The modified Wells-Riley model with indoor CO_^2^_ (C) can be obtained by substituting Equation (2) for Equation (6) as follows:

$PI=\frac{C'}{S}=1-e^{-\frac{C-Co}{M}}\frac{Iqpt}{n}\left ( 1-\eta I \right )\left ( 1-\eta S \right )$　　　　　(7)

$Ro=S\cdot PI$　　　　　(8)

The Ro value was employed to determine an acceptable level of individual exposure risk from a public health perspective, where the prevention of outbreaks is essential. Ro​ refers to the expected number of secondary infections generated by a single infected individual in a completely susceptible population. To reduce the spread of the virus, the target exposure risk level must be set to Ro < 1 [8].

The variables of the adapted Wells-Riley model, incorporating indoor CO_2_ data, include C, Co, M, I, q, p, t, n, ηI, and ηS, as detailed in Table 2. The benchmark for C was established at 1,000 ppm, a level generally recognized in Japan as the threshold between satisfactory and unsatisfactory ventilation. Meanwhile, Co was determined to be 417.9 ppm based on the analysis reported by the World Meteorological Organization on November 15, 2023 [9].

**Table 2:** Parameters for modified Wells-Riley model with indoor CO2 concentration * Refer to the "Models and Data Analysis" section of this paper for information on the generation rate of infectious quanta for measles. CO_2_: carbon dioxide; NDIR: non-dispersive infrared; Ro: basic reproduction number; h: hours

Parameters	References
Atmospheric CO_2_ concentration	Recommend measurement with a CO_2_ monitor employing the NDIR method
Indoor CO_2_ concentration	Recommend measurement with a CO_2_ monitor employing the NDIR method
Exposure time (h)	
CO_2_ emission rate for a person (m^3^/h)	0.011 at rest, 0.01795 for talking^10)^
Generation rate of infectious quanta (/h)	2345 /h*
Pulmonary ventilation rate for a person (m^3^/h)	0.48 for adult male^15)^
Number of infectious individuals	
Number of individuals staying in the room	
Exhalation filtration efficacy	0 for no mask, 0.5 for surgical mask, 0.9 for N95 mask^14)^
Respiration filtration efficacy	0 for no mask, 0.5 for surgical mask, 0.9 for N95 mask^14)^
Results	
Infectious probability (%)	
Basic reproduction number (Ro)	Ro >1 suggests the spread of virus infection^8)^

Tajima et al. observed that a male adult’s CO_2_ emission rate (M) fluctuated between 0.011 and 0.0840, depending on the intensity of physical activity: 0.011 at rest, 0.0129-0.0230 during sedentary work, 0.0230-0.0330 when walking slowly, 0.0330-0.0538 for light labor, 0.0538-0.0840 for moderate labor, and >0.0840 for heavy labor [10]. They recommended adjusting these values by 0.9 and 0.5 for women and children, respectively. Accordingly, we set the CO_2_ emission rates for a male adult in this study.

In the Wells-Riley model, the parameter q is crucial as it reflects the contagiousness of an airborne pathogen based on epidemiological studies. When deriving a new version of the Wells-Riley model, it’s necessary to recalculate the quanta generation rate for the specific pathogen, taking into account the assumptions used in the new risk model. For example, Riley et al. used a steady-state Wells-Riley model to estimate the quanta generation rate for measles during an outbreak in a New York elementary school, finding it to be between 480 and 5,589 quanta per hour [6]. Later, Rudnick and Milton reported a rate of 570 quanta per hour using a different set of assumptions [11]. Additionally, Chen et al. calculated a rate of 128 quanta per hour using a transient model for the same measles outbreak originally studied by Riley et al. [12]. Building on these well-known studies of measles outbreaks in U.S. schools, Azimi et al. derived the quanta generation rate for measles using a newly developed risk model [13]. This new model divided the school environment into three microenvironments: the infector’s classroom, recirculation areas, and common spaces. While most variables were directly reported in the studies by Riley et al. and Chen et al., some parameters were not documented during the outbreaks. In those cases, we used a range of values and selected the best estimate for each parameter. According to this study, the best estimates for the primary and secondary school cases were q = 1,925 (95% CI: 1,185-3,345) and q = 2,765 (95% CI: 1,430-5,140], respectively. Consequently, we adopted an average q value of 2,345 quanta per hour as the quanta generation rate for measles.

Sickbert-Bennett et al. assessed the filtration efficiency of hospital-grade face masks and found that even N95 masks with suboptimal fit achieved over 90% efficacy [14]. In contrast, surgical masks, whether tied or with ear loops, had lower filtration efficiencies ranging from 37% to 69%, due to their thinner filters and looser fit. In our study, the values for ηI and ηS​ were set at 0%, 50%, and 90% for unmasked individuals, surgical masks, and N95 masks, respectively. Table 2 presents the parameters of the adapted Wells-Riley model along with their reference values [15].

## Results

Restaurants

**Figure 1:**
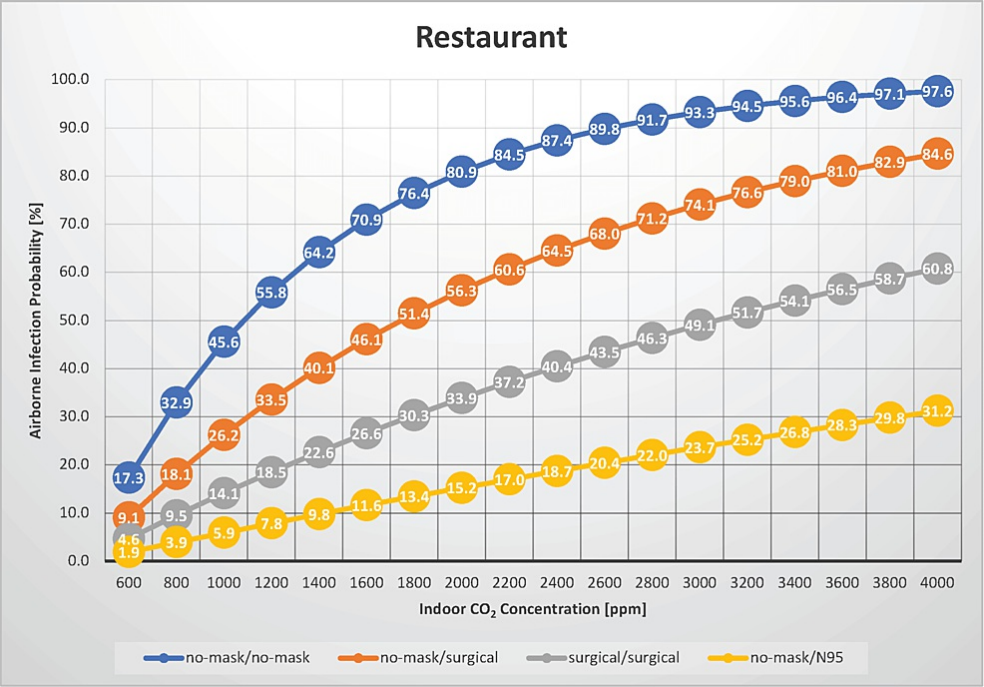
The relationship between indoor CO2 concentration and the probability of airborne transmission of the measles virus in a restaurant CO_2_:_ _carbon dioxide

In a scenario where 30 people were dining and conversing in a restaurant for 30 minutes, the airborne transmission rate of measles was estimated under the indoor CO_2_ concentration ranged from 600 to 4,000 ppm (Figure 1), which could similarly be applied to settings such as class discussions, club activities, and workplace meetings.

Lee et al. conducted a comprehensive investigation into indoor air quality across four restaurants in metropolitan Hong Kong, which included a Korean barbecue restaurant, a Chinese hot pot restaurant, a Chinese dim sum restaurant, and a Western canteen. Their findings indicated that indoor CO_2_ concentrations ranged from 636 to 2,344 ppm [16]. When the indoor CO_2_ concentration is 600 ppm, indicating a well-ventilated environment, and neither the infected individual nor the susceptible individuals are wearing any masks, the airborne transmission rate of measles is 17.3%. Notably, a moderate risk of airborne infection persists even in such well-ventilated conditions. When the indoor CO_2_ concentration is 2,400 ppm, indicating poor ventilation, and neither the infected individual nor the susceptible individuals are wearing any masks, the airborne transmission rate of measles is 87.4%. Compared with well-ventilated conditions, the infection risk increased by >70%. Therefore, maintaining good ventilation is crucial to mitigating the risk of infection. When infected and susceptible individuals wear surgical masks, the risk of airborne transmission can be reduced by up to approximately 45%, depending on the indoor ventilation conditions. Although wearing surgical masks constantly may not be practical in such scenarios, wearing masks during non-eating periods can help mitigate the risk of airborne transmission to some extent. However, the risk of airborne transmission can be reduced to between 1.9% and 31.2% in immunocompromised individuals wearing N95 masks, even when the infected individual is not wearing a mask. In scenarios with good ventilation (indoor CO_2_ concentration <800 ppm), the risk of airborne transmission can be reduced to <5%. Under conditions of an indoor CO_2_ concentration of 1,000 ppm and with neither the infected nor susceptible individuals wearing masks, the Ro is estimated to be 13.2, indicating that restaurants are one of the scenarios where clusters are likely to occur, requiring particular caution.

Live events

**Figure 2:**
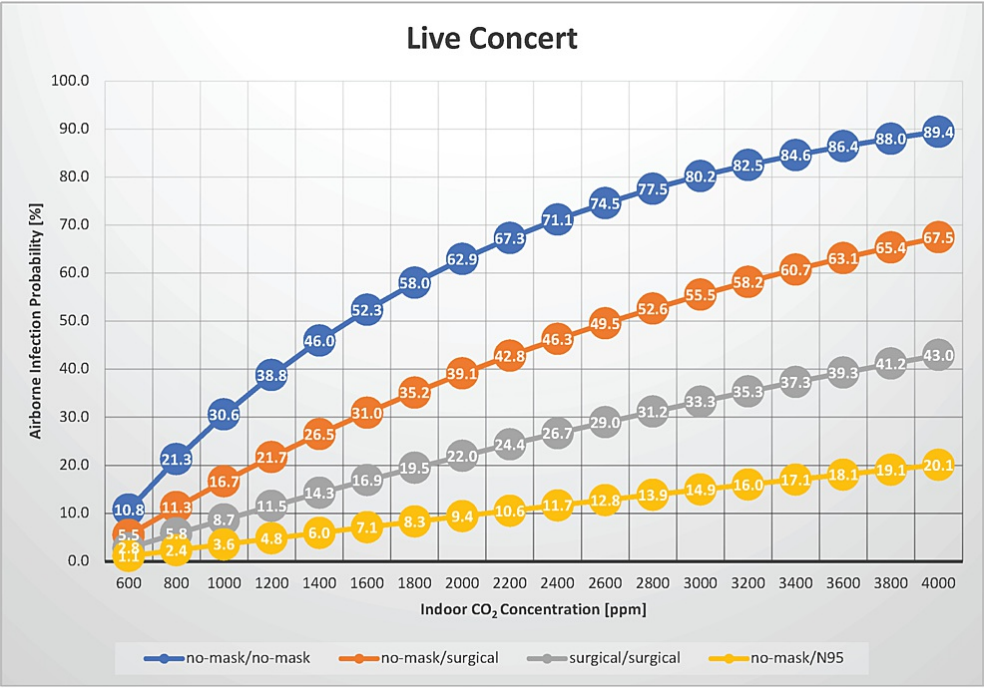
The relationship between indoor CO2 concentration and the probability of airborne transmission of measles virus in a live concert CO_2_: carbon dioxide

In a scenario where 200 people were gathered for a two-hour event, such as a live show where the audience was vocal, the probability of airborne transmission of measles was estimated under indoor CO_2_ concentrations ranging from 600 to 4,000 ppm (Figure 2).

This scenario can also be applied to settings, such as conferences, political rallies, sports events, church services, and school assemblies. Adzic et al. evaluated indoor air quality at mass gathering events and revealed that the CO_2_ concentration in the auditorium of a certain theater, with attendance at 24% of maximum capacity, had average and maximum CO_2_ concentrations of 516 and 618 ppm, respectively. In another theater, with attendance at 94% of maximum capacity, the average and maximum CO_2_ concentrations were 1,211 and 1,617 ppm, respectively [17]. Under conditions of good ventilation with an indoor CO_2_ concentration of 600 ppm and with neither the infected individual nor the susceptible individuals wearing masks, the airborne transmission rate of measles is 10.8%. Even in such well-ventilated environments, a significant risk of infection cannot be ignored. Under conditions of poor ventilation with an indoor CO_2_ concentration of 1,600 ppm and with neither the infected individual nor the susceptible individuals wearing masks, the airborne transmission rate of measles is 52.3%. Compared with well-ventilated conditions, the risk of infection is estimated to increase by over 40%. Therefore, maintaining good ventilation is crucial to reducing the risk of infection. When both the infected and susceptible individuals wear surgical masks, the airborne transmission risk can be reduced by up to approximately 45%, depending on the indoor ventilation conditions. In such scenarios, using surgical masks is recommended. The risk of infection can be reduced in immunocompromised individuals by wearing surgical masks, even if the infected person is not wearing any mask, depending on indoor ventilation conditions. Furthermore, the risk of airborne transmission is reduced to <5% if immunocompromised individuals wear N95 masks, provided the ventilation is relatively good (indoor CO_2_ concentration <1,200 ppm). Under conditions of an indoor CO_2_ concentration of 1,000 ppm and with neither the infected nor susceptible individuals wearing masks, the Ro is estimated to be 60.9, indicating that audience-participatory live concerts are scenarios that require particular caution, as they are prone to the occurrence of clusters.

Home

**Figure 3:**
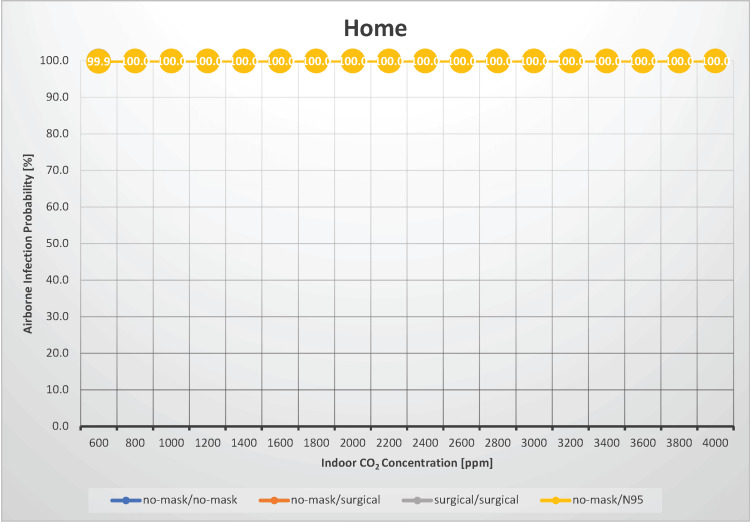
The relationship between indoor CO2 concentration and the probability of airborne transmission of the measles virus at home CO_2_: carbon dioxide

In a scenario where four people stayed at home for 24 hours, the probability of airborne transmission of measles was estimated under indoor CO_2_ concentrations ranging from 600 to 4,000 ppm (Figure 3).

This scenario could be applied to other settings, such as road or boat trips. Schieweck et al. evaluated indoor air quality in smart homes and found that the median indoor CO_2_ concentrations at various measurement points ranged from 587 to 1,360 ppm [18]. However, even with good ventilation conditions and an indoor CO_2_ concentration of 600 ppm, the risk of airborne transmission remains 100%. Regardless of ventilation conditions, the airborne transmission rate is 100%. In such a scenario, ventilation is not a significant factor in determining the risk of airborne transmission. Regardless of the presence or type of masks worn by infected and susceptible individuals, the airborne transmission rate is 100%, indicating that wearing masks to prevent airborne transmission within a household is almost ineffective. Because ventilation and mask-wearing are ineffective in preventing measles transmission within a household, measures such as spatial or temporal isolation of the infected individual can be considered effective strategies for reducing the risk of airborne transmission. Under conditions of an indoor CO_2_ concentration of 1,000 ppm and with neither the infected nor susceptible individuals wearing masks, the Ro is estimated to be 3.0, implying that if one individual was infected with measles in the household, all family members would have contracted the infection the next day. This underscores the significance of household transmission as a crucial scenario for sustaining the spread of infection within the community.

Business meetings

**Figure 4:**
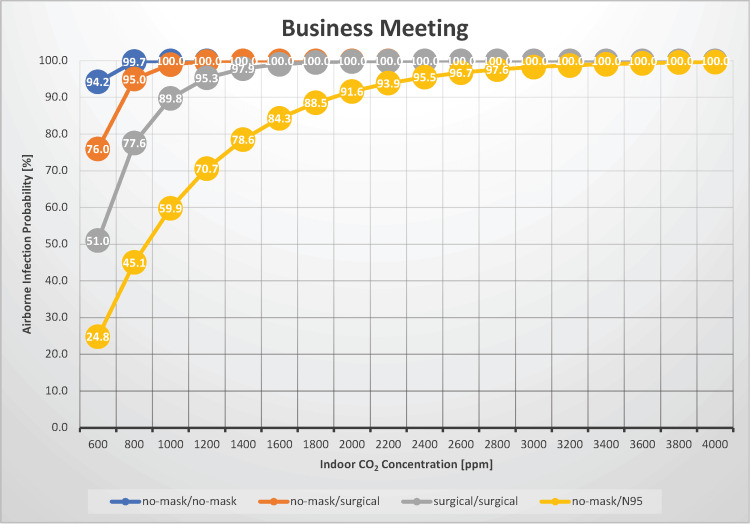
The relationship between indoor CO2 concentration and the probability of airborne transmission of the measles virus in a business meeting CO_2_: carbon dioxide

In a scenario where two people were engaged in a conversation for 30 minutes, such as during a business meeting, the probability of airborne transmission of measles was estimated under indoor CO_2_ concentrations ranging from 600 to 4,000 ppm (Figure 4).

This scenario could also be applied to situations such as job interviews, doctor’s appointments, and study sessions. In our previous study, the indoor CO_2_ concentrations in hospital outpatient settings, with five occupants per room, served as a reference. The median CO_2_ concentration in pediatric and respiratory outpatient rooms was 1,116 and 549 ppm, respectively [5]. Under conditions of good ventilation with an indoor CO_2_ concentration of 600 ppm and with neither the infected individual nor the susceptible individual wearing masks, the airborne transmission rate of measles is 94.2%, indicating that even in well-ventilated environments, the occurrence of an airborne infection is very highly likely. Under conditions of poor ventilation with an indoor CO_2_ concentration of 2,000 ppm and with neither the infected nor the susceptible individual wearing masks, the airborne transmission rate of measles is 100%, making ventilation not a significant factor that determines the risk of airborne transmission. When both the infected and susceptible individuals wear surgical masks, the risk of airborne transmission can be reduced by up to approximately 45%, depending on the indoor CO_2_ concentration. Therefore, in such scenarios, the proactive use of surgical masks is recommended. Moreover, immunocompromised individuals can reduce the risk of airborne transmission by up to approximately 20% by wearing a surgical mask, compared with scenarios where the infected individual is not wearing any mask. Furthermore, if immunocompromised individuals wear N95 masks, the risk of airborne transmission can be reduced by up to approximately 70%. However, even under the best ventilation conditions (indoor CO_2_ concentration of 600 ppm), a moderate airborne transmission risk of 24.8% remains. Under conditions of an indoor CO_2_ concentration of 1,000 ppm and with neither the infected nor susceptible individuals wearing masks, the Ro is estimated to be 1.0, implying a significant likelihood of airborne transmission occurring during a business meeting with a measles-infected individual.

## Discussion

Airborne infection probability comparison with SARS-CoV-2

**Figure 5:**
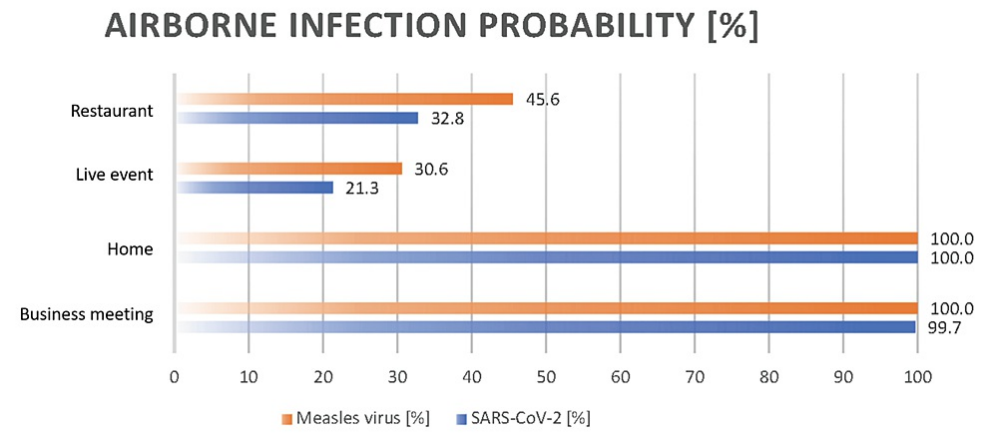
Comparison of the airborne transmission probability of the measles virus with SARS-CoV-2 under 1,000 ppm indoor CO2 concentration across four scenarios CO_2_: carbon dioxide

We examined the importance of indoor ventilation in various scenarios using the difference in the probability of the airborne transmission of the measles virus at indoor CO_2_ concentrations of 600 and 4,000 ppm as an indicator of ventilation efficacy (Figure 5).

In the absence of surgical masks for infectious and susceptible individuals and under an indoor CO_2_ concentration of 1,000 ppm, the respective differences in airborne transmission probability for each scenario were 45.6%, 30.6%, 100%, and 100% in restaurants, live events, at home, and in business meetings, respectively.

**Figure 6:**
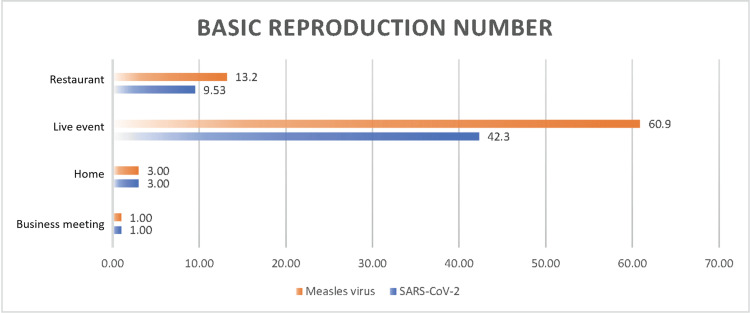
Basic reproduction number of the measles virus with SARS-CoV-2 under 1,000 ppm indoor CO2 concentration across four scenarios CO_2_: carbon dioxide

SARS-CoV-2 was identified as a novel coronavirus in December 2019 in Wuhan, Hubei Province, China. It is the causative agent of COVID-19, a novel viral infection [19]. Based on WHO data, as of June 9, 2024, 775,615,736 cases of COVID-19 were confirmed, and 7,051,323 deaths were confirmed globally [20]. Figure 6 compares the airborne transmission rates and Ro of SARS-CoV-2 and the measles virus.

These data indicate that both the airborne transmission rate and Ro of the measles virus are higher than those of SARS-CoV-2. Consequently, in the event of a measles pandemic, the number of infected individuals would surpass that of a SARS-CoV-2 outbreak. In the future, if signs of a measles pandemic emerge, implementing stringent measures will be crucial in high-risk environments, such as restaurants and live events. These measures should include the mandatory wearing of masks and ensuring good ventilation. Additionally, individuals who have encountered an infected person in settings, such as homes or business meetings, should be treated as close contacts and subjected to strict temporal and spatial isolation to mitigate the risk of transmission.

We investigated four representative scenarios, with Table 1 showing the parameters for each outline. In the four scenarios presented, the airborne transmission probability of the measles virus can be easily predicted by using a CO_2_ concentration meter alongside the graphs provided in this study. Carbon dioxide concentration meters are available on the market with varying degrees of accuracy; however, we recommend using a meter employing the nondispersive infrared method for higher precision. Notably, these parameters may vary slightly depending on the circumstances. Therefore, inserting context-specific parameters into Equation (7) is necessary to estimate accurate airborne transmission probabilities and basic reproduction numbers.

Limitations

This study has several limitations. First, the Wells-Riley model assumes that the air within a room is thoroughly mixed, ensuring a uniform aerosol distribution, indicating that the model addresses airborne transmission but not droplet or contact transmission. Consequently, the modified Wells-Riley model, incorporating CO_2_ levels, should not be used in scenarios with predominant droplet or contact transmission. Second, this model is appropriate solely for steady-state conditions and is inapplicable in areas with significant movement of individuals. Finally, the model is unsuitable for outdoor environments due to its reliance on indoor CO_2_ concentration. Additionally, the research showed significant variability regarding the quanta generation rate (q), a crucial factor in determining the airborne transmission rate of measles. The value of q = 2345/h was adopted in this study and is not an absolute figure. This value is likely representative of the quanta generation rate during conversations, but the rate during nonconversational scenarios remains unknown. Consequently, the airborne transmission risk can be estimated in nonconversational situations, such as school classes, commuter trains, or concert halls. Further research is needed to address these gaps.

## Conclusions

Our comprehensive study, which utilized the modified Wells-Riley equation to assess the airborne transmission risk of the measles virus in diverse indoor environments, has provided crucial insights into infection control strategies. Graphical representations for each scenario were utilized to predict measles virus transmission rates using CO_2_ concentration meters. We have ascertained that CO_2_ monitoring, when used as an indirect indicator of ventilation efficacy, is a valuable tool in mitigating the risk of airborne virus transmission. Therefore, this study advocates for a multifaceted approach that integrates CO_2_ monitoring, mask usage, and ventilation enhancements to prevent the spread of the measles virus indoors. Moreover, it offers a framework for risk assessment in various settings and supports informed decision-making in the development of public health policies.

## References

[1] (2024). Measles and rubella. https://www.who.int/westernpacific/health-topics/measles.

[2] (2024). Western Pacific countries at risk of measles outbreaks due to immunization and surveillance gaps. https://www.who.int/westernpacific/news/item/01-03-2024-western-pacific-countries-at-risk-of-measles-outbreaks-due-to-immunization-and-surveillance-gaps.

[3] (2024). Chapter 13: measles. DC: Public Health Foundation.

[4] Fine PE (1993). Herd immunity: history, theory, practice. Epidemiol Rev.

[5] Iwamura N, Tsutsumi K (2023). SARS-CoV-2 airborne infection probability estimated by using indoor carbon dioxide. Environ Sci Pollut Res Int.

[6] Riley EC, Murphy G, Riley RL (1978). Airborne spread of measles in a suburban elementary school. Am J Epidemiol.

[7] Dai H, Zhao B (2020). Association of the infection probability of COVID-19 with ventilation rates in confined spaces. Build Simul.

[8] Yan S, Wang LL, Birnkrant MJ, Zhai J, Miller SL (2022). Evaluating SARS-CoV-2 airborne quanta transmission and exposure risk in a mechanically ventilated multizone office building. Build Environ.

[9] 9] World Meteorological Organizaiton (WMO) (2023, November 15 (2024). WMO Greenhouse Gas Bulletin No. 19 - 15 November 2023: the state of greenhouse gases in the atmosphere based on global observations through 2022. https://library.wmo.int/records/item/68532-no-19-15-november-2023.

[10] Tajima M, Inoue T, Ohnishi Y (2016). Estimation of occupants’ carbon dioxide production rate for measurement of ventilation (Article in Japanese). J Environ Eng AIJ.

[11] Rudnick SN, Milton DK (2003). Risk of indoor airborne infection transmission estimated from carbon dioxide concentration. Indoor Air.

[12] Chen SC, Chang CF, Liao CM (2006). Predictive models of control strategies involved in containing indoor airborne infections. Indoor Air.

[13] Azimi P, Keshavarz Z, Cedeno Laurent JG, Allen JG (2020). Estimating the nationwide transmission risk of measles in US schools and impacts of vaccination and supplemental infection control strategies. BMC Infect Dis.

[14] Sickbert-Bennett EE, Samet JM, Clapp PW (2020). Filtration efficiency of hospital face mask alternatives available for use during the COVID-19 pandemic. JAMA Intern Med.

[15] Liao CM, Chang CF, Liang HM (2005). A probabilistic transmission dynamic model to assess indoor airborne infection risks. Risk Anal.

[16] Lee CS, Li WM, Chan LY (2001). Indoor air quality at restaurants with different styles of cooking in metropolitan Hong Kong. Sci Total Environ.

[17] Adzic F, Roberts BM, Hathway EA (2022). A post-occupancy study of ventilation effectiveness from high-resolution CO(2) monitoring at live theatre events to mitigate airborne transmission of SARS-CoV-2. Build Environ.

[18] Schieweck A, Uhde E, Salthammer T (2018). Smart homes and the control of indoor air quality. Renew Sustain Energy Rev.

[19] Guan WJ, Ni ZY, Hu Y (2020). Clinical characteristics of coronavirus disease 2019 in China. N Engl J Med.

[20] (2024). Coronavirus disease (COVID-19) pandemic. https://www.who.int/emergencies/diseases/novel-coronavirus-2019.

